# Neurological Complications Associated with Respiratory Syncytial Virus Infections: A Scoping Review of Prospective Clinical Trials Conducted in Populations up to 17 Years of Age

**DOI:** 10.3390/pathogens14050503

**Published:** 2025-05-20

**Authors:** Aikaterini S. Stravoravdi, Xanthippi Topalidou, Georgios Papazisis

**Affiliations:** Clinical Research Unit, School of Medicine, Aristotle University of Thessaloniki, 54624 Thessaloniki, Greece; kate_st@outlook.com (A.S.S.); xanthippitopalidou@gmail.com (X.T.)

**Keywords:** encephalopathy, neurological complications, respiratory syncytial virus, prospective clinical trials, scoping review

## Abstract

Objective: This study aimed to review the neurologic and cognitive complications of respiratory syncytial virus (RSV) infections through studies involving infants, children, and adolescents up to 17 years of age. Methods: The PubMed, Scopus, Cochrane Library, and PsycINFO databases were comprehensively searched for prospective clinical trials written in the English language that assess the neurologic and/or cognitive complications of RSV. This review was conducted according to the recommendations of the PRISMA-ScR checklist. Results: The vast majority of studies have concentrated on younger children, with limited investigation into long-term cognitive outcomes. While data on adolescents are sparse, this likely reflects the lower incidence of RSV-related neurological complications in this age group, rather than a critical gap in the research. The key findings from the 11 included studies highlighted a range of neurologic complications, which are particularly prevalent in children under six years of age. One study reported delayed language development and potential memory impairments, highlighting the virus’s potential impact on early cognitive processes. Conclusions: The findings of this review highlight the need for more clinical studies focusing on the impact of RSV on the central nervous system and potential complications that may arise, particularly in groups at high risk of acquiring this infection. Future investigations should focus on longitudinal assessments to elucidate long-term effects and standardize methodologies for enhanced comparability.

## 1. Introduction

Respiratory syncytial virus (RSV) is a leading cause of acute lower respiratory tract infections in infants and young children worldwide. While the majority of RSV infections are mild or asymptomatic and resolve without medical intervention, a small proportion of cases—particularly among infants, immunocompromised patients, or those with underlying conditions—may lead to severe illness requiring hospitalization [1]. The significant morbidity and mortality in these age groups has, in recent years, increased the pressing need for vaccination against RSV, mainly targeting children, the elderly, and pregnant women, and two vaccines were recently approved internationally [2].

Growing evidence indicates that in rare instances, neurological manifestations associated with RSV infection have been reported, including febrile seizures; encephalopathy; and, less frequently, encephalitis or meningoencephalitis. However, it is important to note that RSV is not considered a major cause of central nervous system (CNS) infections compared to more prominent etiologic agents such as enterovirus or parechovirus [3,4,5]. Febrile seizures, while among the most commonly observed neurological events in children with RSV, are typically non-specific, are not indicative of direct CNS invasion, and resolve spontaneously without complications. Common infections that trigger febrile seizures include human herpes virus type 6 (HHV-6) and influenza, while RSV is less commonly associated with this presentation. Considering that almost all children are infected with RSV during the first three years of life (and many suffer from more than one infection), the role of RSV in febrile seizures is less important than those of some other infections [6,7]. Finally, although some studies report that central apnea may occur in a subset of newborns with RSV infections—with variable prevalence estimates—such events are uncommon and typically limited to specific age groups or clinical contexts. In light of these considerations, this review aimed to systematically explore and synthesize the available literature on the neurologic and cognitive manifestations of RSV infections in the pediatric population while acknowledging the rarity of these outcomes and the need for cautious interpretation of the current evidence base.

The underlying biological mechanisms of these central nervous system (CNS) manifestations remain unclear and possibly involve both direct viral invasion and indirect immune-mediated responses. RSV has been detected in the cerebrospinal fluid (CSF) of infected individuals, indicating its ability to breach the blood–brain barrier and directly infect the CNS. Animal studies have demonstrated the presence of RSV RNA and proteins in various brain regions, including the hippocampus and cortex, following intranasal infection. This direct infection correlates with observed behavioral and cognitive impairments, such as deficits in spatial learning and memory, suggesting that RSV can have long-term effects on brain function [8,9]. Elevated levels of pro-inflammatory cytokines and chemokines, such as IL-6, IL-8, CCL2, and CCL4, have been observed in patients with RSV-related neurological symptoms. These molecules can be produced by activated astrocytes and microglia in response to infection, leading to neuroinflammation. This inflammatory response can contribute to neuronal dysfunction and damage, potentially resulting in encephalopathy and other CNS complications [10].

Although recent systematic reviews have been published on the occurrence of CNS complications due to RSV [3], to the best of our knowledge, there is a gap in the literature regarding a focused review of well-designed prospective clinical trials in younger populations. Therefore, the aim of this scoping review was to explore the literature for prospective clinical trials involving infants, children, and adolescents up to 17 years of age, specifically addressing neurological and cognitive complications associated with respiratory syncytial virus infection.

## 2. Materials and Methods

The neurologic and cognitive complications of RSV represented the aim of this review. The scoping review methodology was chosen to meet the research objective of mapping the current evidence on the wide-ranging topic of neurologic and cognitive complications following RSV infection. This approach also sought to outline key features of the included studies—particularly their designs—in order to accommodate variability and inform directions for future research. This scoping review was conducted according to the recommendations of the PRISMA-ScR checklist (Preferred Reporting Items for Systematic Reviews and Meta-Analyses extension for Scoping Reviews) [11]. The protocol is registered on the International Platform of Registered Systematic Review and Meta-analysis Protocols (registration number: INPLASY202510017, DOI number: 10.37766/inplasy2025.1.0017INPALSY).

### 2.1. Inclusion and Exclusion Criteria

Studies reporting results in infants, children, and adolescents up to 17 years of age were considered eligible. Any neurological or cognitive outcome potentially associated with a positive RSV infection was considered an outcome of interest. Language and study type restrictions were applied; only articles published in English and prospective clinical trials were included. Other types of trials were excluded, as well as reports referring to the peripheral and autonomic nervous systems. Studies that did not report neurological or cognitive outcomes were excluded. Eligible studies included prospective observational cohort or case series studies, while case reports and case series with fewer than five patients were excluded. While central apnea may occur in some infants with RSV, particularly newborns, it is generally considered a respiratory or mixed pathophysiological event rather than a primary neurological complication and thus was not included within the scope of this review [12]. Due to the current inability to reliably differentiate between central and obstructive apnea, apnea was not categorized as a neurological complication for the purposes of this review. Therefore, studies specifically focused on apnea outcomes were excluded. No additional restrictions were applied.

### 2.2. Search Strategy

To identify relevant studies, MEDLINE (via PubMed), Scopus, the Cochrane Library, and PsycINFO were queried. The research was last updated on 26 November 2024. The search algorithm included a combination of the keywords and their synonyms, which are presented in the Supplementary Materials.

### 2.3. Data Analysis and Synthesis

Articles not written in English were excluded to ensure linguistic consistency for analysis. Titles and abstracts were screened for relevance, and full-text control was applied for all potentially eligible studies by one author with confirmation by a second author. Duplicate articles were removed. Two studies described a common subset of the same patients, and in this case the most recent study was included. Data from the included studies were extracted into a Microsoft Word document in the form of a table. All data on neurological or cognitive outcomes, regardless of the initial reporting methods in the included studies, were extracted. Additionally, the number of participants and the population age distribution were outcomes of interest. Narrative synthesis of the data was then performed, describing the current knowledge derived from prospective trials in relation to the expression of respiratory syncytial virus in the central nervous system. The findings are presented in full in a tabular format.

## 3. Results

### The Results of the Literature Search

**Figure 1:**
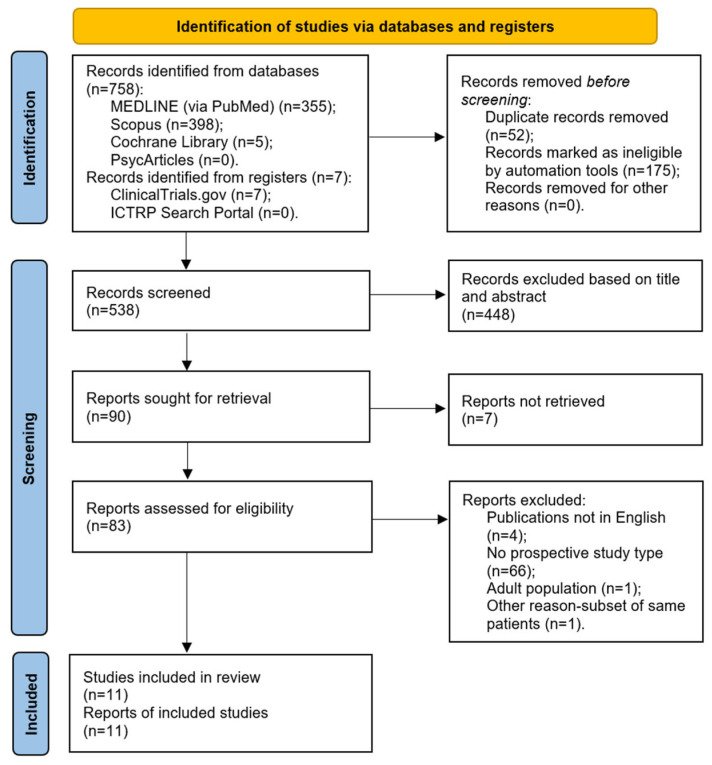
A PRISMA 2020 flow diagram.

The initial database search yielded 765 reports. After removing duplicates and ineligible entries using an automated tool in Scopus, 538 records were available for screening. The title and abstract review led to the exclusion of 448 records that were unrelated to the research question. Seven additional records could not be retrieved. The remaining 83 reports underwent full-text screening for eligibility. Of these, one was excluded for involving an adult population, and another was removed due to the data overlapping with a previously included study. In addition, 4 non-English articles and 66 studies that did not meet the design criteria—such as retrospective studies, case reports, and systematic reviews—were also excluded. In total, 11 articles fulfilled the inclusion criteria and were included in the final analysis. The study selection process is depicted in a PRISMA flow diagram (Figure 1). A summary of the included studies is presented in Table 1.

The prospective study conducted by Savić et al. [13] between November 2008 and March 2009 reported the clinical manifestations of RSV infection in children under 12 months of age. In total, 15 out of 91 participants (16.5%) developed complications, including apnea, significant atelectasis, and encephalopathy. The authors did not provide specific information regarding the clinical symptoms and signs associated with these complications.

Nygaard et al. [14] researched clinical severity factors in RSV-positive children up to the age of 5 years during the 2021–2022 RSV season and the pre-COVID-19 RSV seasons (2016–2020), differentiating outcomes for children with and without risk factors. The study design was divided into a retrospective analysis of the pre-COVID-19 seasons and a prospective analysis of the 2021–2022 RSV season. The retrospective data of this study are not presented in the current review. According to the results, a total of 54 children required assisted ventilation support during the 2021–2022 RSV period. More than half of these children (n = 35) were categorized as being at high risk of severe RSV disease, while the remaining 19 children had no risk factors for severe disease. The reported rates of neurological complications among these children were 14% in the high-risk group and 21% in the group without risk factors. Prolonged or complex febrile seizures were documented in three children (16%) in the group of participants without risk factors, while the corresponding number in the high-risk group was three (9%). One child in each group developed acute encephalopathy/encephalitis (5% in the group without risk factors and 3% in the group with risk factors). In the high-risk group, one case (3%) of hyponatremia-related seizures was reported. Overall, a substantial percentage of the patients required intubation and mechanical ventilation due to CNS complications, with most cases occurring in patients without risk factors for severe disease.

The EFES multicenter study [15], conducted between March 2016 and April 2017, enrolled 174 children aged 2 to 60 months who presented with febrile seizures. The majority of the episodes (69.5%) were characterized as simple febrile seizures, with a recurrence rate of 41.4% for febrile seizures. Viral detection from nasopharyngeal samples was performed to identify potential viral causality. RSV was identified in 16% of the positive nasopharyngeal swabs. RSV B was more prevalent, detected in 9.7%, whereas RSV A was identified in 6.25% of the positive nasopharyngeal swabs. Furthermore, a higher prevalence of RSV A was connected to simple febrile seizures, a finding characterized by statistical significance (*p* < 0.05). Specifically, RSV B as a sole infectious agent was identified in four cases (2.7%), while RSV A was identified in one case (0.7%), with the remaining RSV identifications involving coinfections with other viruses. The most common viruses among the positive nasopharyngeal swabs in the study population were adenovirus, influenza A and B, and rhinovirus. HHV-6, a known neurotropic virus associated with febrile seizures [16], was not included in the virological identification panel of the EFES study.

An additional prospective trial reported by Erez et al. [17], conducted between 2011 and 2012, enrolled and analyzed 14 infants with RSV-positive infections and symptoms with central nervous system involvement. Most of the infants experienced central apneas, whereas three presented with encephalopathy. No evidence of RSV RNA positivity was found in the cerebrospinal fluid (CSF) samples. The authors emphasize that this finding does not support direct viral replication in the CNS, suggesting that further mechanisms should be investigated.

**Table 1:** The studies included in the review.

Study	Key Results: Neurological and Cognitive Outcomes	Study Population
Savić et al., 2011 [13]	RSV complications: apnea, significant atelectasis and/or encephalopathy in 15 patients (16.5%)	n = 91 children (<12 months)
Nygaard et al., 2023 [14]	RSV-related complications requiring mechanical ventilation (2021–2022): Children with no risk factors (n = 19): Any neurological complication: 4 (21%); Prolonged or complex febrile convulsions: 3 (16%); Acute encephalopathy or encephalitis: 1 (5%); Hyponatremia-associated convulsions: 0. Children with risk factors (n = 35): Any neurological complication: 5 (14%); Prolonged or complex febrile convulsions: 3 (9%); Acute encephalopathy or encephalitis: 1 (3%); Hyponatremia-associated convulsions: 1 (3%).	Children (0–17 years)
Carman et al., 2018 [15]	Identification of RSV in 16% of positive nasopharyngeal swabs in patients with febrile seizures (RSV B: 9.7%, RSV A and B: 6.25%)	n = 174 children (2–60 months)
Erez et al., 2014 [17]	Reports of encephalopathy (clinical somnolence): 3 No identification of RSV in CSF (RSV antigen-positive in rapid enzyme-linked immunoassay)	n = 14 infants (<12 months)
Kawashima et al., 2012 [10]	The following outcomes were reported: Excitotoxic encephalopathy (n = 6); Hypoxic encephalopathy (n = 1); Cytokine-storm encephalopathy (n = 1); Unconsciousness (n = 8); Generalized seizures and partial seizures (n = 1); Generalized seizures only (n = 3); Generalized seizures after cardiopulmonary arrest (n = 1); Cerebellar ataxia and nystagmus (n = 1); Clonic seizures after cyanosis (n = 1); Partial seizure (n = 1); Brain edema on CT (n = 6); Mental retardation (n = 2); Quadriplegia (n = 1).	n = 8 children (10 days–3 years)
Peña et al., 2020 [18]	Significant difference, favoring the control group, regarding the first phrases, words, and gestures that the infants understood, said, or used at 12 months (*p* = 0.03)	n = 89 infants (<15 months)
Jiang et al., 2023 [19]	Clinical neurological complications of RSV patients that needed ICU therapy (pre-pandemic and during the pandemic in 2021): Meningitis: 4 (1.1%) in the pre-pandemic period (n = 376), 1 (1.5%) in 2021 (n = 66) (*p* = 0.56); Seizures: 4 (1.1%) in the pre-pandemic period (n = 376), 2 (3.0%) in 2021 (n = 66) (*p* = 0.22).	Children in different age groups (<15 years)
Pokorn et al., 2017 [20]	Prevalence of RSV in NS and stool in patients with febrile seizures (n = 192 for NS and n = 165 for stool samples): 10.9 (6.9–16.2) (%, 95% CI) Prevalence of RSV in NS and stool in healthy controls (n = 156 for NS and n = 150 for stool samples): 1.3 (0.2–4.6) (%, 95% CI) OR adjusted for age (95% CI) = 7.2 (2.2–36.7) (*p* < 0.001) Proportion (%) and number of RSV-positive patients with simple FS: 11.4 (15/132) Proportion (%) and number of RSV-positive patients with complex FS: 10.0 (6/60) OR for complex FS in virus-positive patients (95% CI): 0.9 (0.3–2.3) (*p* = 0.838)	n = 192 children (<6 years)
Tang et al., 2014 [21]	n = 9 (4.8%) RSV-positive children in the febrile seizure group n = 31 (17.8%) RSV-positive children in the control group	n = 363 children (6 months–6 years)
Wilkesmann et al., 2007 [22]	Seizures in children hospitalized with RSV infection: NMI group (n = 73): 15; Control group (n = 1495): 2 (*p* < 0.001).	n = 1541 pediatric patients
Hautala et al., 2021 [23]	RSV identification in all patients (n = 145): 8%. RSV in patients 6 months–3 years: Febrile seizures: 10 (6%); No febrile seizures: 90 (8%) (*p* = 0.291). RSV in patients 3 years–6 years: Febrile seizures: 5 (11%); No febrile seizures: 40 (8%) (*p* = 0.284).	n = 1899 children (6 months–6 years)

RSV: respiratory syncytial virus, CSF: cerebrospinal fluid, CT: Computer tomography, ICU: intensive care unit, NS: nasopharyngeal swab, CI: Confidence Interval, OR: odds ratio, FS: febrile seizures, NMI: neuromuscular impairment.

A total of eight children up to 3 years old with confirmed RSV infections and CNS symptoms were investigated by Kawashima et al. [10]. Six cases of excitotoxic encephalopathy, one case of hypoxic encephalopathy, and one case of cytokine-storm encephalopathy were reported, with a variety of clinical presentations. Most patients experienced generalized convulsions, including one case following cardiopulmonary arrest. Additionally, cases of cerebellar ataxia, cyanosis followed by clonic seizures, and a partial seizure were each reported in a single patient. Mental retardation was an outcome in two patients, and quadriplegia was reported in one patient. The diagnostic criteria regarding the outcome of mental retardation were not further specified in the methodology of this study.

Peña et al. [18] investigated the impact of severe RSV infection on learning ability in 89 infants. Difficulties in acquiring native phonemes and communicating at 1 year of age were identified, with a statistically significant difference observed in the infants’ vocabularies and comprehension at 12 months. The authors highlight potential disorders affecting the process of linguistic development and the possibility of memory impairments.

In a long-term prospective clinical trial conducted between 2016 and 2022, reported by Jiang et al. [19], the clinical manifestations of RSV infection were investigated. For this study, children and adolescents up to the age of 16 years were eligible patients. The data were presented separately for the pre-pandemic period and the COVID-19 period. A total of 376 RSV-positive patients required intensive care unit (ICU) therapy between 2016 and 2020. In the pandemic year of 2021, 66 patients were admitted to the ICU. Meningitis occurred at rates of 1.1% in the pre-pandemic patients and 1.5% in the patients admitted during 2021. RSV infection was complicated by seizures in 1.1% of participants in the pre-pandemic period, and this rate increased by 3% in the 2021 cohort. Apnea was reported as a complication at rates of 3.2% for the patients in the pre-pandemic period and 3% for the patients in 2021.

In the study by Pokorn et al. [20], the RSV positivity rate among other viruses was tested in children under 6 years of age with febrile seizures, with healthy children serving as a control group. The RSV detection rate was, among others, an outcome of the study, which aimed to compare this rate between children admitted to the hospital with febrile seizures and those admitted for elective surgical procedures. A statistically significant difference in RSV rates was observed between the participants with febrile seizures and the healthy controls. Specifically, the RSV-positive nasopharyngeal samples from the patients with febrile seizures were detected at a rate of 10.9%, compared to 1.3% in the healthy participants, with an age-adjusted odds ratio (OR) of 7.2. Additionally, the detection rate of the virus in patients with simple febrile seizures was 11.4%, and in cases of complex febrile seizures it was 10%.

Another prospective study, published by Tang et al. [21], included children with febrile seizures within the age spectrum of 6 months to 6 years, and participants were enrolled between 2010 and 2011. In this study, common respiratory viruses, including RSV, were detected in children with upper respiratory tract infections. Specifically, the active group included children with febrile seizures and upper respiratory tract infections, whereas the control group consisted of children with upper respiratory tract infections and fevers but without seizures. The study results indicated that RSV was detected in 4.8% of patients with febrile seizures, compared to 17.8% in the control group, which was statistically significant. Outcomes related to the seizures were reported for both the influenza and non-influenza groups of the study, but no additional specific information for RSV can be extracted from the results.

Wilkesmann et al. [22] conducted a multicenter prospective study that enrolled RSV-infected children over six seasons from 1999 until 2005. The participants were categorized into two groups: children with and without known neuromuscular impairments. The group of participants with existing neuromuscular impairments experienced seizures at a significantly higher rate, with 15 out of 73 patients reporting seizures, compared to two cases of seizures among the 1495 participants without pre-existing neurological conditions.

The study by Hautala et al. [23] included children 6 months to 6 years of age and assessed RSV incidence in two groups of patients: those with and without febrile seizures. A total of 145 study participants tested positive for RSV. Among children aged 6 months to 3 years, RSV was detected in 6% of the patients presenting with febrile seizures and 8% of those without febrile seizures. In the age group of 3 years to 6 years, the rates were 11% and 8%, respectively. RSV was identified in 5% of the cases with simple febrile seizures, compared to a 10% incidence in cases of complex febrile seizures, with a relative risk of 1.56.

## 4. Discussion

This scoping review summarized the study characteristics reported in the reviewed literature for the neurologic and cognitive manifestations of RSV through studies involving infants, children, and adolescents up to 17 years of age. Global data presented by the World Health Organization (WHO) indicate that RSV is related to roughly 3.6 million hospitalizations annually. RSV-related mortality can reach up to 100.000 cases per year among children up to five years of age, with the majority occurring in low- and middle-income countries [24]. According to data from the U.S. population presented by The Respiratory Syncytial Virus Hospitalization Surveillance Network, the age group of 0–4 years has been associated with the highest rates of hospitalizations among all age groups in all RSV seasons since 2018–2019. It is noteworthy that the age group of 5–17 years has generally low hospitalization rates [25].

Overall, the analyzed studies differed in the age ranges of the populations they included. While some focused on infants and young children up to 3 years of age, others encompassed a broader range from 6 months to 6 years. A few studies also included older children. Only one study involved adolescents up to 16 years of age [19]. This inconsistency in age criteria across studies presents challenges for making direct comparisons. However, with respect to RSV, this review identified only one published study available on cognitive manifestations [18]. There was a statistically significant difference in the infants’ vocabulary and comprehension at 12 months, indicating that they had trouble learning local phonemes and communicating at 1 year of age. Attention was drawn to possible memory deficits and diseases related to the language development process. There was considerable heterogeneity among the included studies in terms of how outcomes were measured and reported. This variation stemmed from differences in study design: some were epidemiological studies investigating viral causes of febrile convulsions in children, while others focused specifically on neurological outcomes as complications of RSV infection. One study exclusively examined neurodevelopmental outcomes, while another compared seizure incidence between RSV-infected children with and without pre-existing neurological conditions. As a result, the absence of standardized approaches to measuring neurologic and cognitive outcomes poses significant challenges for informing clinical decision-making.

The neurologic and cognitive manifestations of RSV infection in children and adolescents represent a complex and multifaceted challenge in pediatric healthcare. RSV, a leading cause of acute respiratory infections, often leads to severe complications, particularly in younger populations. Manifestations such as febrile seizures, apnea, encephalopathy, and developmental delays highlight this virus’s potential to impact the CNS directly or indirectly. Early-life RSV infections frequently result in lower respiratory tract symptoms, conditions that are often associated with neurologic outcomes like seizures and altered consciousness [13,14]. Evidence suggests that mechanisms such as inflammation, cytokine responses, or hypoxia may underlie these neurologic effects, as findings regarding direct viral invasion of the CNS remain inconsistent [10,17].

### 4.1. Neurologic Manifestations

A range of studies have documented the neurologic complications linked to RSV. Savić et al. [13] documented complications such as apnea, significant atelectasis, and encephalopathy in 16.5% of infants under 12 months, underscoring the clinical challenges posed by severe RSV cases. However, this study was not designed to reveal the neurologic complications of RSV infections. Therefore, robust clinical correlations cannot be established. Nygaard et al. [14] reported febrile seizures and encephalopathy among children during the 2021–2022 RSV season, noting a significant prevalence of seizures, even among children without predisposing risk factors. This unpredictability highlights RSV’s potential for severe neurologic consequences across diverse clinical profiles. Notably, in the study population, children not considered to be high-risk for severe RSV disease had a higher incidence of neurologic complications compared to those without risk factors. However, the number of participants was insufficient to draw significant conclusions.

Studies such as those by Kawashima et al. [10] and Wilkesmann et al. [22] reported more severe neurologic outcomes, including excitotoxic and cytokine-storm encephalopathy, seizures, and even permanent disabilities like quadriplegia and mental retardation. Children with pre-existing neuromuscular impairments experienced significantly higher rates of seizures compared to their healthy counterparts [22]. Similarly, Jiang et al. [19] investigated long-term trends in RSV complications, observing a marked increase in neurologic symptoms during the COVID-19 pandemic compared to earlier years. The intersection of RSV dynamics and pandemic-altered healthcare likely exacerbated these neurologic outcomes.

### 4.2. Cognitive and Developmental Outcomes

The cognitive and developmental impacts of RSV were explored in fewer studies, but the findings remain critical. Peña et al. [18] identified significant delays in language acquisition and phoneme recognition among infants with severe RSV infections. These results suggest that RSV may disrupt early neurodevelopmental processes, potentially resulting in long-term cognitive deficits. The cognitive dimension of RSV infection, although underexplored, aligns with concerns about broader neurodevelopmental sequelae and their implications for affected children’s academic and social trajectories.

### 4.3. Febrile Seizures

The association between RSV and febrile seizures was a recurring theme. The EFES study [15] linked RSV infections with febrile seizures in children aged 2–60 months, identifying RSV A as significantly associated with simple febrile seizures. However, in this study, other viruses such as adenovirus, influenza A and B, and rhinovirus were identified at much higher rates in the patients’ positive nasopharyngeal swabs. Additionally, the number of participants was insufficient to support robust clinical suggestions. Pokorn et al. [20] similarly found higher rates of RSV detection among children presenting with febrile seizures compared to healthy controls, emphasizing the virus’s potential role in febrile seizure pathogenesis. Hautala et al. [23] and Tang et al. [21] provided additional evidence for this association, with their results highlighting RSV’s role across both simple and complex febrile seizure cases. These findings reinforce the need for careful monitoring of febrile seizures as a potential marker of RSV complications.

### 4.4. Mechanistic Insights

The mechanism of RSV-induced neurologic manifestations remains a critical area of inquiry. Erez et al. [17] found no RSV RNA in cerebrospinal fluid samples from infants with central apnea and encephalopathy, suggesting that inflammatory responses or cytokine-driven processes, rather than direct CNS invasion, may mediate these symptoms. This hypothesis is supported by the diverse presentations reported in studies like that by Kawashima et al. [10], where cytokine-storm encephalopathy and hypoxic events were observed.

The mechanisms by which respiratory syncytial virus affects the central nervous system are not yet fully elucidated, but several plausible pathways have been proposed. One theory involves direct viral invasion of the CNS. Although RSV is primarily a respiratory pathogen, viral RNA has occasionally been detected in cerebrospinal fluid and brain tissue, suggesting the potential for hematogenous dissemination or transneuronal spread via peripheral nerves such as the olfactory or vagus nerve. The detection of RSV in the cerebrospinal fluid of infected individuals suggests it has the ability to breach the blood–brain barrier and directly infect the CNS [8,9]. However, direct CNS invasion appears to be an uncommon event. More frequently, RSV-related neurological complications are believed to be mediated by systemic immune and inflammatory responses. Severe RSV infection triggers elevated levels of pro-inflammatory cytokines, which can cross the blood–brain barrier and promote neuroinflammation. This cytokine-mediated mechanism has been implicated in the development of encephalopathy, seizures, and other neurologic symptoms, even when direct viral presence in the CNS cannot be demonstrated [26].

### 4.5. Limitations

The included studies are characterized by heterogenous design methods and reporting strategies, leading to variability in the reported results. A direct and robust synthesis of all the data is challenging since there are no common outcomes in most of the studies. An additional limitation is the lack of evidence observed in the field of CNS complications after RSV infection, specifically in relation to prospective data collection from patients. The current data do not demonstrate with sufficient analytical power that RSV is a driver of febrile seizure disorder any more than any other cause of rapid fever onset and thus do not add to the urgency of vaccine prevention on that score. Relatively small sample sizes limit the generalizability of the results and complicate the interpretation of the relationship between the virus and potential neurological symptoms. As this was a scoping review, we did not perform a risk-of-bias assessment, consistent with the scoping review methodology. While this limited our ability to assess the quality of the included studies, the objective of the current review was to provide an overview of the existing literature rather than evaluate the strength of the evidence. Additionally, potential underestimation of the CNS involvement in mild RSV cases is a limitation since many studies focused on complications in patients that needed ICU therapy. Cognitive and behavioral symptoms may have been underreported at a significant rate, particularly because specific assessment tools were not used by individual studies and the follow-up periods did not allow long-term observation. A language bias may also be present, as only articles in English and German were considered eligible.

## 5. Conclusions

This scoping review explored the neurologic and cognitive manifestations of RSV infections in the pediatric population, highlighting critical findings and identifying research gaps, particularly in younger children, where the burden of disease is greatest. This review also uncovered limited evidence on cognitive manifestations, such as language delays and potential memory deficits, with only one study addressing these outcomes in depth. These findings align with the review’s objectives, offering insights into the complex interplay between RSV and CNS involvement.

Neurological manifestations were frequently associated with severe RSV infections, with studies emphasizing the indirect pathogenic mechanisms underlying CNS complications, such as excitotoxicity and immune-mediated damage. The significant association between febrile seizures and RSV, particularly RSV A, underscores the need for heightened clinical vigilance in diagnosing and managing affected children. However, the scarcity of studies addressing adolescents and long-term cognitive outcomes presents a challenge, highlighting the need for more inclusive research to fully understand the age-related effects of RSV.

### Implications and Next Steps

Our findings emphasize that long-term cognitive outcomes following RSV infection remain significantly underrepresented in the literature. Future research should address these gaps by focusing on (i) conducting longitudinal studies to assess long-term cognitive and developmental impacts, particularly in infants and young children, who represent the most affected population; (ii) standardizing methodologies and outcome measures across studies to improve the comparability and generalizability of findings; and (iii) expanding studies to include broader pediatric populations, paying careful attention to age-stratified analyses where appropriate.

While evidence for significant neurological involvement in adolescents is limited, age-specific studies may still provide useful insights into variations in immune responses, risk factors for complications, and recovery trajectories across developmental stages. However, research prioritization should continue to be guided by the observed clinical burden.

Notably, only one study in this review extensively addressed cognition and language outcomes following RSV infection, underscoring a significant gap in the current evidence base. The potential for mild neurologic or cognitive impairments, which may remain undetected in short-term or routine clinical follow-ups, warrants greater attention. Subtle deficits in executive function, language development, attention, or processing speed could have meaningful implications for a child’s academic performance and quality of life but may only become apparent through more sensitive, standardized neurodevelopmental assessments conducted over extended follow-up periods. Future prospective studies should incorporate comprehensive, age-appropriate cognitive, language, and behavioral evaluations using validated instruments to capture a broader spectrum of neurodevelopmental outcomes.

In conclusion, this review highlights the importance of collaborative, multidisciplinary efforts to advance our understanding and improve outcomes for children affected by RSV. By gaining deeper insights into RSV’s neurologic and cognitive effects, these efforts will enhance clinical care and guide public health strategies aimed at reducing the global impact of this common pediatric infection.

## Data Availability

Data sharing is not applicable.

## Supplementary Materials

The following supporting information can be downloaded at https://www.mdpi.com/article/10.3390/pathogens14050503/s1, Document S1: Search algorithm.
