# Supplementary material for: Neurological Complications Associated with Respiratory Syncytial Virus Infections: A Scoping Review of Prospective Clinical Trials Conducted in Populations up to 17 Years of Age
Source: Pathogens. 2025 May 20;14(5):503. doi: 10.3390/pathogens14050503 (PMC12114428; doi:10.3390/pathogens14050503)
Supplement: Supplementary file 1 [file pathogens-14-00503-s001.zip › pathogens-3595168-supplementary.pdf]

PubMed Search Algorithm:

("Respiratory Syncytial Viruses"[MeSH Terms] OR ("respiratory"[All Fields] AND "syncytial"[All Fields] AND "viruses"[All Fields]) OR ("Respiratory Syncytial Viruses"[All Fields] OR ("respiratory"[All Fields] AND "syncytial"[All Fields] AND "virus"[All Fields]) OR ("respiratory syncytial virus"[All Fields] OR ("syncytial"[All Fields] AND "virus"[All Fields] AND "respiratory"[All Fields]) OR ("syncytial virus respiratory"[All Fields] OR ("syncytial"[All Fields] AND "viruses"[All Fields] AND "respiratory"[All Fields]) OR ("virus"[All Fields] AND "respiratory"[All Fields] AND "syncytial"[All Fields]) OR ("viruses"[All Fields] AND "respiratory"[All Fields] AND "syncytial"[All Fields]) OR "viruses respiratory syncytial"[All Fields])))) AND ("Central Nervous System"[MeSH Terms] OR ("central"[All Fields] AND "nervous"[All Fields] AND "system"[All Fields]) OR "Central Nervous System"[All Fields] OR "Central Nervous System"[All Fields] OR "neurological"[All Fields] OR "neurological manifestation"[All Fields] OR "neurological complication"[All Fields] OR "brain"[All Fields] OR "spinal cord"[All Fields] OR "cerebral"[All Fields] OR "neurologic"[All Fields] OR "encephalopathy"[All Fields] OR "seizures"[All Fields] OR "convulsion"[All Fields] OR "convulsions"[All Fields] OR "neurologic disease"[All Fields])

Scopus Search Algorithm:

(TITLE-ABS-KEY (cns) OR TITLE-ABS-KEY ("central nervous system") OR TITLE-ABS-KEY ("cognitive impairment") OR TITLE-ABS-KEY ("central nervous system dysfunction") OR TITLE-ABS-KEY (encephalitis) OR TITLE-ABS-KEY (encephalopathy) OR TITLE-ABS-KEY (seizures) OR TITLE-ABS-KEY ("neurological disorders") OR TITLE-ABS-KEY (neurological AND disease) AND TITLE-ABS-KEY ("respiratory syncytial virus infection")) AND (LIMIT-TO (OA , "all"))
